# Sex-Specific Modulation of the Host Transcriptome in the Spleen of *Schistosoma mansoni*-Infected Mice

**DOI:** 10.3389/fcimb.2022.893632

**Published:** 2022-07-05

**Authors:** Franziska Winkelmann, Anne Rabes, Cindy Reinholdt, Nicole Koslowski, Dirk Koczan, Emil C. Reisinger, Martina Sombetzki

**Affiliations:** ^1^ Department of Tropical Medicine and Infectious Diseases, Center of Internal Medicine II, Rostock University Medical Center, Rostock, Germany; ^2^ Institute of Immunology, University of Rostock, Rostock, Germany

**Keywords:** *Schistosoma mansoni*, unisexual infection, transcriptome analysis in spleens, host–parasite interaction, immunomodulation

## Abstract

**Background:**

Schistosomiasis is a severe parasitic disease that is primarily driven by the host’s immune response to schistosome eggs trapped in tissue and by the granulomatous inflammatory and fibrotic reaction they cause. Despite significant progress in understanding the complex immunological processes involved in the relationship between schistosomes and their host, neither an effective vaccine against the infection nor anti-fibrotic drugs currently exists, making the search for new targets for schistosome drugs and vaccine candidates even more important. In order to identify new molecular targets for defense against or elimination of the parasite, we investigate herein the interplay between the host and male or female schistosomes, clearly separating this from the action of the parasite eggs.

**Methods:**

For this purpose, we infected 6–8-week-old female NMRI mice with 100 male (M), female (F), or both (MF) *S. mansoni* cercariae and performed a comparative transcriptomic and flow cytometric analysis of their spleens.

**Results:**

Principal component analysis of a total of 22,207 transcripts showed a clear clustering of the experimental groups. We identified a total of 1,293 genes in group M, 512 genes in group F, and 4,062 genes in group MF that were differentially expressed compared to naive controls. The highest percentage of regulated genes (2,972; 65.9%) was found in group MF alone, but there was a large overlap between groups M and MF (798; 17.7%) and a small overlap between groups F and MF (91; 2.0%). Only 4.5% of genes (201) were revealed to be regulated in all experimental groups (M/F/MF). In addition, we were able to show that both worm sexes trigger immune responses in an egg-independent manner (non-polarized Th1 and Th2 response), with female worms exerting less regulatory influence than males.

**Conclusion:**

Our data show that adult schistosomes trigger sex-specific, egg-independent immune responses. The lists of genes regulated by adult female or male worms presented here may be useful in deciphering host–parasite interactions to identify targets for schistosome elimination.

## Introduction

Schistosomiasis remains one of the most common parasitic tropical diseases of the 21st century, affecting more than 230 million people worldwide and causing an estimated 1.9 million disability-adjusted life years (DALYs; Weerakoon et al., 2015; Hay et al., 2017). It is a severe parasitic disease caused predominantly by the host immune response to tissue-trapped schistosome eggs, and by the granulomatous inflammatory and fibrotic reaction which they trigger. In the absence of a reliable vaccine to control the spread of the disease, current treatment strategies, including mass drug administration, rely heavily on the anthelmintic praziquantel (PZQ; WHO, 2022). However, the therapy is insufficient because PZQ is ineffective against developmental stages and fails to prevent reinfection. In addition, mass treatments in the course of containment efforts may cause resistance to develop (Fallon and Doenhoff, 1994). Therefore, interest in schistosomiasis research is clearly driven by the pressure to find a vaccine against the *Schistosoma* spp. in order to prevent reinfections in endemic areas, particularly in children, and to limit transmission.

Humans and other vertebrates become infected with the parasite *via* contaminated freshwater. The infective larval stages, which are free swimming in the water, penetrate the skin and mature on their way through the lungs, heart, and liver to become adult worms which finally mate in the portal vein. From here, they migrate as worm pairs, the male carrying the female in its ventral cavity, into the venous plexuses (mesenteric or pelvic) of their target organs, intestine or bladder. Schistosomes exhibit pronounced sexual dimorphism. *Schistosoma mansoni* is the most widespread member of the genus (Gryseels et al., 2006). It remains in the mesenteric vessels for the duration of its lifetime, and the eggs deposited in the blood by female worms are washed retrogradely by the bloodstream to the liver, causing severe hepatosplenic disease. Soluble egg antigens trigger vigorous granulomatous inflammation which leads to hepatic fibrosis and thus to impaired hepatic blood flow, portal hypertension, bleeding from esophageal varices, and ascites (Stavitsky, 2004; Burke et al., 2009). The inflammation process corresponds to a Th2 immune response that, later in the chronic course of the disease, changes to a Th2/Treg response. As well as egg antigens, antigens of the gonochoric adult worms circulate in the blood and are also thought to trigger immune responses (Wilson et al., 2015; Cosenza-Contreras et al., 2018). Adult worms are equipped with an amazing variety of mechanisms to evade the host’s immune attacks (Angeles et al., 2020), enabling them to survive for decades in the hostile environment of their host (Gryseels et al., 2006).

The immunostimulatory molecules of adult worms are diverse and circulate as antigens in the bloodstream of the host. As early as 1953, an experiment with rhesus monkeys showed that an initial infection with male schistosomes resulted in immunity upon subsequent bisexual challenge infection (Vogel and Minning, 1953; Hsü, 1969). In unisexual infection, male worms are significantly more immunogenic than females and induce a stronger immune response in the murine host (Boissier et al., 2003). In a previous study, we have shown that female schistosomes can suppress the host’s early immune response to invading cercariae and trigger an upregulation of anergy-associated genes. Moreover, a primary infection with female *S. mansoni* cercariae resulted in a reduced Th2 response upon subsequent bisexual infection, as evidenced by smaller liver granulomas and less pronounced liver fibrosis (Koslowski et al., 2017). Male schistosomes, on the other hand, elicit a strong innate immune response which in the livers of experimentally infected mice resulted in a visible reduction in worm and egg burden during unisexual reinfection (Sombetzki et al., 2018).

Little is known about the differences in splenic activity caused by male and female schistosomes, however (Cosenza-Contreras et al., 2018). In this study, we performed a comparative transcriptomic and flow cytometric analysis of spleen tissue from unisexually and bisexually infected mice, additionally examining the immunostimulatory capacity of splenocytes in order to dissect the mechanisms underlying the differential immunogenicity of female and male worms.

## Materials and Methods

### *S. mansoni* Infection Model

*S. mansoni* (Belo Horizonte strain) was kept in a life cycle using *Biomphalaria glabrata* fresh water snails as intermediate hosts and 6–8-week-old female NMRI mice as definitive hosts, as previously described (Sombetzki et al., 2016). To obtain either male or female cercariae with which to subsequently infect the mice, *B. glabrata* snails were exposed to single *S. mansoni* miracidia and cercariae were harvested 6 weeks later. The sex of the cercariae was determined by DNA amplification of sex-related chromosome segments using female-specific primers as previously described (Koslowski et al., 2017).

To bring about infection, 6-week-old female C57BL/6 mice were percutaneously exposed to 100 *S. mansoni* cercariae, either male only (M), female only (F), or both sexes (MF). Controls were left untreated (naive). Mice were sacrificed 4 or 8 weeks p.i. (*n* = 6–8) *via* cervical dislocation under isoflurane anesthesia. The spleens were weighed, related to body weight, and collected for further analysis.

### RNA Extraction and Quality Control

RNA extraction was performed on the spleens of mice (*n* = 2–3) which had been infected 8 weeks previously using the RNeasy Plus Kit (Qiagen, Hilden, Germany). The procedure included a DNA removal step according to the manufacturer’s protocol. Before the protocol was applied, the spleen in its entirety was homogenized in liquid nitrogen using a cryo mortar and a pestle with the addition of 1ml chaotropic buffer (RLT Plus) from the RNeasy Plus Kit (Qiagen, Hilden, Germany). The tissue/buffer powder was transferred to a 1.5-ml reaction tube and, after thawing, an aliquot of 50 μl was taken and diluted with 550 μl RNeasy Plus buffer for the cleanup process. Finally, a phenol/chloroform extraction (phenol/chloroform/IAA, 25:24:1, pH 6.6; Thermo Fisher Scientific, Waltham, MA, USA) was performed. This procedure enabled us toaverage the expression pattern of all spleen substructures and ensure that we did not overload the RNeasy spin column. The whole RNA samples were quantified spectrophotometrically (Nanodrop 1000, Thermo Fisher Scientific) and diluted to a concentration of 70 ng/μl. RNA integrity was tested using an Agilent RNA 6000 Nano Chip with a Bioanalyzer 2100 instrument (Agilent Technologies). The samples achieved RNA integrity numbers between 8.6 and 9.8.

### Microarray Hybridization

A 200ng whole RNA sample was used as starting material in the GeneChip^R^ Whole Transcript Sense Target Labeling protocol (Affymetrix, St. Clara, CA, USA). Microarray hybridization was carried out using the AffymetrixClariom™ S Array Kit according to the manufacturer’s instructions (Affymetrix, St. Clara, CA, USA). In brief, the “Whole Transcriptome” protocol starts with first strand synthesis by introducing T7 promoter tags to all RNA molecules using N6 3’ ends. After strand replacement according to Eberwine, non-labeled antisense RNA (aRNA) is produced by *in vitro* transcription in concert with the linear amplification of all RNA molecules without a 3’ bias. After a magnet bead-based cleanup of the aRNA, a new strand-identical single-strand DNA is produced by adding random primers and deoxyribonucleoside 5’-triphosphates (dNTPs) using the aRNA as a template. In the meantime, a certain amount of dTTP is replaced by dUTP. A removal of the aRNA *via* RNase H digestion and a magnet bead-based cleanup, an enzymatic endpoint fragmentation was possible. An uracyldeglycosidase removes the uracils and APE1 (apurinic apyrimidinic endonuclease 1) cleaves the deuracylized phosphodiester backbone of the single strand DNA molecules. A desoxynucleotidyl transferase adds DNA labeling reagent (Biotin-11-dXTP) to the 3’ ends of the single strand DNA fragments. Hybridization was carried out overnight (16 h) at 45°C in the GeneChip^R^ Hybridisation Oven 645 (Affymetrix). Washing and staining protocols including an antibody amplification were carried out by the GeneChipFluidis Station 450 (Affymetrix). The microarrays were scanned using the GeneChip Scanner 3000 7G (Affymetrix) at 0.7-micron resolution.

### Data Processing, Filtering, and Bioinformatics Analysis

AffymetrixClariom™ S Arrays (mouse) interrogate more than 22,100 genes in around 800,000 probes. The generated data (expression values of genes) were analyzed using the Transcriptome Analysis Console (TAC) version 4.0.2.15 (Thermo Fisher Scientific) with the SST-RMA normalization algorithm (robust multi-array average, improved by “signal space transformation”). Technical replicate groups were summarized using LIMMA (linear models for microarray data) statistics. Differentially expressed genes (DEG) were identified using filter parameters, fold change >2 or ≤2, old-change > 2 or ≤ 2, LIMMA p value < 0.05, and FDR q value < 0.05. For the comparative analysis, we always compared groups of infected (M, F, or MF) mice to the group of uninfected mice (naive). Multivariate analysis was carried out by principal component analysis (PCA), also using Transcriptome Analysis Console (TAC) version 4.0.2.15 (Thermo Fisher Scientific). Differential gene expression levels were visualized using TAC version 4.0.2.15 to prepare volcano plots. ShinyGO (version 0.61; Ge et al., 2020) was used to classify genes according to Gene Ontology (GO) categories and analyze them for enrichment. Further, expression levels of chosen genes linked to the host’s immune response were grouped according to their function and visualized using Origin (version 2021; OriginLab) to create a heat map.

### Gene Expression Analysis

Total RNA was isolated from spleen tissue (see RNA extraction) and reversely transcribed into cDNA using High-Capacity cDNA Reverse Transcriptase Kit (Thermo Fisher Scientific, Waltham, MA, USA) according to the manufacturer’s instructions. Real-time PCR was performed using the following TaqMan Gene Expression Assays: *Tlr5* Mm00546288_s1; *CD209a* Mm00460067_m1; *Clec4g* Mm01212425_m1; *Ccl24* Mm00444701_m1; *Epb4.2* Mm00469111_m1; *Cdc20* Mm00650983_g1 (Thermo Fisher Scientific, Waltham, MA, USA). Gene expression values were normalized to the endogenous reference gene *Gapdh* (Rodent GAPDH control reagent, Thermo Fisher Scientific, Waltham, MA, USA) and presented as normalized expression values relative to naive controls.

### Spleen Cell Preparation

Single-cell suspensions were prepared by passing the spleens of mice (*n* = 4–8) infected for 8 weeks through a cell strainer (100 µm) followed by PBS washing and erythrocytes lysis with RBC lysis buffer (BioLegend, San Diego, CA, USA). Cells were washed twice with PBS and cell numbers were quantified using a CASY TT cell counter (OLS-Omni Life Science).

### Flow Cytometry

Cells were stained with Zombie Red™ Fixable Viability Kit (BioLegend, San Diego, CA, USA) for 15 min in PBS at RT followed by incubation with appropriate fluorochrome-conjugated antibodies for 20 min in FACS buffer (PBS + 3% FCS) at 4°C. The following antibodies (BioLegend, San Diego, CA, USA) were used: myeloid panel (anti-CD11b-APC, anti-CD11c-Alexa488, anti-F4/80-PE-Cy7, anti-CD86-BV605, anti-SiglecF-PerCP-Cy5.5, and anti-Ly6G-APC-Cy7) and lymphoid panel (anti-CD19-Alexa488, anti-CD3-APC, anti-CD4-PerCP-Cy5.5, and anti-CD8-PE-Cy7). After washing, flow cytometric analysis was performed using FACS Aria™ IIIu (BD Bioscience) and data were analyzed using FlowJo software (v10.0.7, Tree Star). Live cells were differentiated by gating on the following cell populations: eosinophils (CD11b^+^/SiglecF^+^), neutrophils (CD11b^+^/Ly6G^+^), dendritic cells (CD11c^+^), macrophages (CD11b^+^F4/80^+^), B cells (CD19^+^), T-helper (Th) cells (CD3^+^/CD4^+^), and cytotoxic T cells (CTLs; CD3^+^/CD8^+^).

### Cytokine ELISAs

To assess cytokine production, isolated splenocytes of mice (*n* = 5–8) infected for 8 weeks were cultured in RPMI 1640 medium supplemented with 10% FCS, 25 mM HEPES, and antibiotics. Cells were stimulated by an in-house-generated 10 μg/ml *S. mansoni* soluble worm antigen preparation containing eggs (SWAP and SEA) for 72 h at 37°C. In cell-free supernatants, cytokines were quantified according to the manufacturer’s protocol using DuoSet ELISA Kits (R&D Systems, Minneapolis, MN, USA) detecting INF-γ, IL-13, IL-4, or IL-10.

### Statistics

Statistical analysis was performed using GraphPad Prism 9.0 (GraphPad Software). Values are expressed as mean ± SEM. Differences between groups were analyzed by Kruskal–Wallis test followed by a Dunn’s correction, *p* values < 0.05 were considered statistically significant. *p < 0.05, **p < 0.01, ***p < 0.001, ****p < 0.001.

## Results

### Unisexual Infection With Female or Male *Schistosoma mansoni* Leads to Significant Enlargement of the Spleen

**Figure 1:**
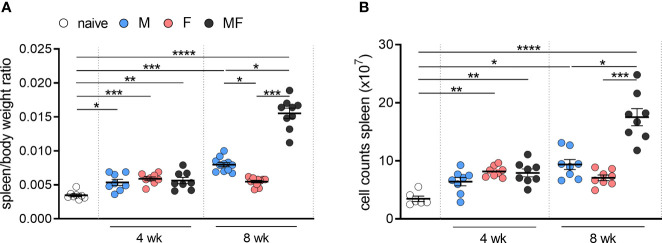
Unisexual infection with female and male *Schistosoma mansoni* leads to significant enlargement of the spleen. Quantification of **(A)** specific weight and **(B)** total cell counts in the spleens of infected (M, F, and MF) and control mice (naive) over time (4 and 8 wk p.i., *n* = 6–8). Data are presented as mean ± SEM. p values < 0.05 were considered statistically significant. *p < 0.05, **p < 0.01, ***p < 0.001, ****p < 0.001; M, infected with male cercariae; F, infected with female cercariae; MF, infected with male and female cercariae.

To analyze the effects of unisexual and bisexual infection with *S. mansoni* on the changes in the spleen, we first quantified the weight of and the total number of cells in the spleens of infected and control mice over time. Compared with naive control mice, infected mice displayed significantly enlarged spleens and higher cell numbers (**Figure 1A, B**). During early infection (4 weeks p.i.), spleen weight and cell numbers did not differ in mice infected with exclusively male (M), exclusively female (F), or male and female (MF) worms. However, during the course of infection (8 weeks p.i.), spleen weight is significantly increased in group M and MF mice compared to group F mice. Spleen enlargement was most pronounced in bisexually infected mice. The data clearly show that the infection-associated enlargement of the spleen is caused not only by the eggs but also by the worm itself, especially male worms.

**Figure 2:**
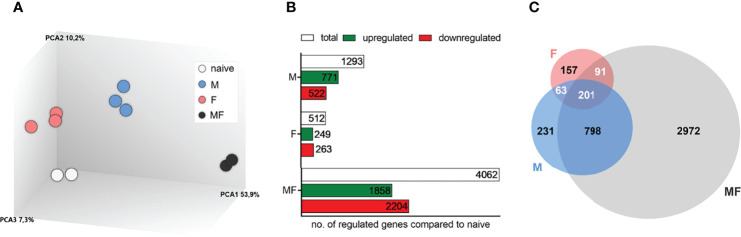
Transcriptomic analysis of spleens of mice uni- and bisexually infected with *Schistosoma mansoni*. Transcriptomic analysis of spleens reveals different gene expression profiles after infection with *S. mansoni* (8 wk p.i.). **(A)** Principal component analysis, **(B)** bar chart, and **(C)** Venn diagram represent an overview of transcriptomic analysis of spleens (*n* = 2–3). Inclusion of all genes showing altered gene expression compared to controls (fold change >2 or à2, LIMMA p value < 0.05, and FDR q value < 0.05); M, infected with male cercariae; F, infected with female cercariae; MF, infected with male and female cercariae.

Transcriptomic analysis of spleens was performed at 8 weeks p.i. to detect differences in the gene expression patterns following infection with male (M), female (F), or male and female *S. mansoni* cercariae (MF) compared to uninfected controls. Principal component analysis (PCA) of a total of 22,207 transcripts showed a clear separation of groups and clustering of individuals in each group (**Figure 2A**). In the spleen tissue, we identified a total of 1,293 genes in group M, 512 genes in group F, and 4,062 genes in group MF that were differentially expressed compared with naive control mice (**Figure 2B**). Comparing the gene sets, we found the majority of regulated genes (2,972; 65.9%) in bisexually infected mice, while 5.1% (231) and 3.5% of genes (157) were exclusively identified following unisexual male (M) and female (F) infection, respectively (**Figure 2C**). The Venn diagram further illustrates that there is a large consensus of genes that were regulated in both M and MF mice (798; 17.7%), whereas the overlap of genes in F and MF groups was relatively small (91; 2.0%). Only 4.5% of genes (201) were regulated in all experimental groups (M, F, MF).

**Figure 3:**
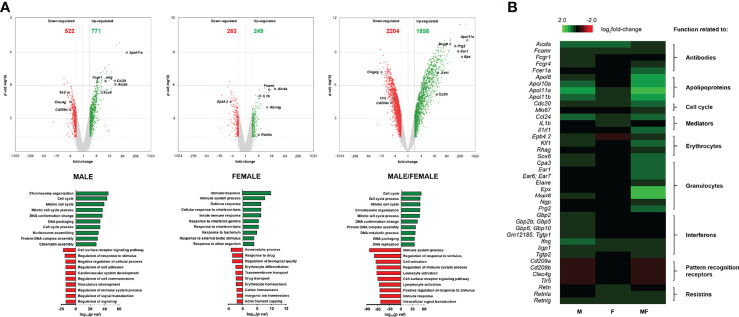
Volcano plot and heatmap of differentially expressed genes in spleen tissue of mice unisexually and bisexually infected with *Schistosoma mansoni*. **(A)** Volcano plots (upper panel) visualizing differentially expressed genes of infected (unisexual and bisexual) mice compared to healthy controls (fold change >2 or ≤2, LIMMA p value < 0.05“. and „Heat map of highly regulated genes related to the host immune response in splenic tissue of infected (M, F, MF)“ into „Heat map of highly regulated genes related to the host immune response in splenic tissue of infected (M, male cercariae; F, female cercariae; MF, male and female cercariae) and FDR *q* value <0.05) plotted against the significance level (negative log10 *p* value) and showing significantly (*p* < 0.05) increased (green) and decreased (red) genes in the spleen. Highly regulated genes are encircled in black. Gene ontology analysis (lower panel) displaying enrichments of biological processes of up- and downregulated genes after infection with *S. mansoni* (8 wk p.i.) compared to naive mice. The ten most enriched categories of biological processes associated with the regulated genes are presented. **(B)** Heat map of highly regulated genes related to the host immune response in splenic tissue of infected (M, F, MF) compared to control (naive) mice (8 wk p.i., *n* = 2–3). The color scale on the top illustrates the log2 fold change values shown in the heat map. Upregulated genes are highlighted in green whereas downregulated genes are marked red. The baseline data are shown in **Supplementary Table 1**.

To further investigate the biological processes occurring in the spleen of *S. mansoni*-infected mice compared with naive mice, gene ontology analysis of up- and downregulated genes was performed using the filtered transcriptomic data (**Figure 3A**). Overall, the enrichment of biological processes of up- and downregulated genes was strong in group MF compared with naive mice following infection with *S. mansoni*, while only minor enrichment was detected in group F. Most of the upregulated genes in groups M (771 genes) and MF (1,858 genes) were associated with cell cycle processes, chromosome organization, and DNA packaging. These results are consistent with our previous observations of increased cell number in these groups (**Figure 1B**). In contrast, group F mice showed enrichment of 249 upregulated genes for processes related to immune response, particularly interferon-dependent mechanisms. In contrast, the MF group displayed the most significant enrichment in the 2,204 downregulated genes related to the immune system and involved in regulatory processes or immune cell activation. In line, the 522 downregulated genes of group M are also mostly associated with regulatory processes that are linked to the immune system as well. The main gene ontology terms for the 263 downregulated genes of group F are homeostatic processes primarily involving erythrocytes, drug response, and transport processes. Overall, the transcriptomic data show that bisexual infection with *S. mansoni* had the strongest effects on gene expression in the spleen compared to naive or unisexually infected mice. However, unisexual infection also shows significant sex-specific differences at the level of gene expression, with group M showing high concordance with group MF.While groups M and MF downregulate genes related to regulatory processes of the immune system, group F increases the expression of genes related to the immune response.

### Unisexual Infection Results in the Upregulation of Genes Related to Host Immune Response

To unravel the mechanisms and pathways initiated by the interplay between the host and the male or female schistosomes, we examined in depth highly regulated genes directly or indirectly associated with the host immune response. Candidates of interest were categorized, and their gene expression listed and visualized using a heat map (**Supplementary Table 1**; **Figure 3B**). Upregulated genes are highlighted in green and downregulated genes are highlighted red. Overall, bisexual infection with *S. mansoni* (MF) has the strongest effect on the expression of genes in our selection belonging to the host immune response compared to naive animals. However, strong immune regulatory effects were also found in cases of unisexual infection (M and F). In the category “Antibodies,” we found *activation-induced cytidine deaminase* (*aicda*; fold changes: +27.0 and +20.4) and genes coding for Fc receptors (*fcamr*, *fcgr1*, *fcgr4*; fold changes: +11.0, +4.2, +10.4 and +11.4, +2.1, +2.7) among the most strongly upregulated genes in unisexually infected mice. Genes encoding “Apolipoproteins” (e.g., *apol8*, *apol10a*, *apol11a*, *apol11b*), which are involved in host plasma lipid adsorption, were upregulated in a range between 5.4- and 87.7-fold in group M mice and between 2.06- and 2.52-fold in group F mice. Additionally, genes related to “Cell cycle” processes were upregulated in group M (*cdc20*, *mki67*; fold changes: +7.6, +4.6). This is in line with the enrichment of genes associated with cell cycle processes in the gene ontology analysis (**Figure 3A**) as well as enhanced cell counts in this group (**Figure 1B**). In the category “Mediators,” there was a 25.3-fold upregulation of the gene coding for CCL24 in group M. In contrast, in group F, we detected a 4.5-fold induction of the pro-inflammatory cytokine IL-1ß. Genes associated with “Erythrocytes” were found to be induced following unisexual male infection. Genes of transcription factors necessary for erythrocyte development (e.g., *sox6*, *klf1*, *gata1*) and erythrocyte-expressed proteins (e.g., *epb4.2*, *rhag*, *ank1*) were upregulated 6.6- to 2.4-fold in group M mice. However, *epb4.2* was downregulated 4.5-fold in group F mice. For genes of the category “Interferons,” we detected in group M a 9.9-fold induction of *interferon gamma* (*ifng*) and upregulation of other IFNG-stimulated genes such as interferon-inducible GTPases (*iigp1*, *igtp*, *tgtp1*, *tgtp2*; fold changes: +11.8, +9.3, +9.6, +8.3) and interferon-induced guanylate-binding proteins (*gbp2*, *gbp2b/5*, *gbp10/6*; fold changes: +8.8, +3.9, +6.4). In the category “Resistins,” all genes (*retn*, *retnla*, and *retnlg*) are upregulated in group F mice (foldchanges: +2.4, +3.8, +9.3), while in group M only *retnlg* is upregulated (fold change: +6.2). On closer examination, we found genes of host “Pattern recognition receptors” such as *cd209a*, *cd209b*, *clec4g*, and *tlr5* to be downregulated in group M (fold changes: -3.8, -5.3, -3.7, -4.6).

**Figure 4:**
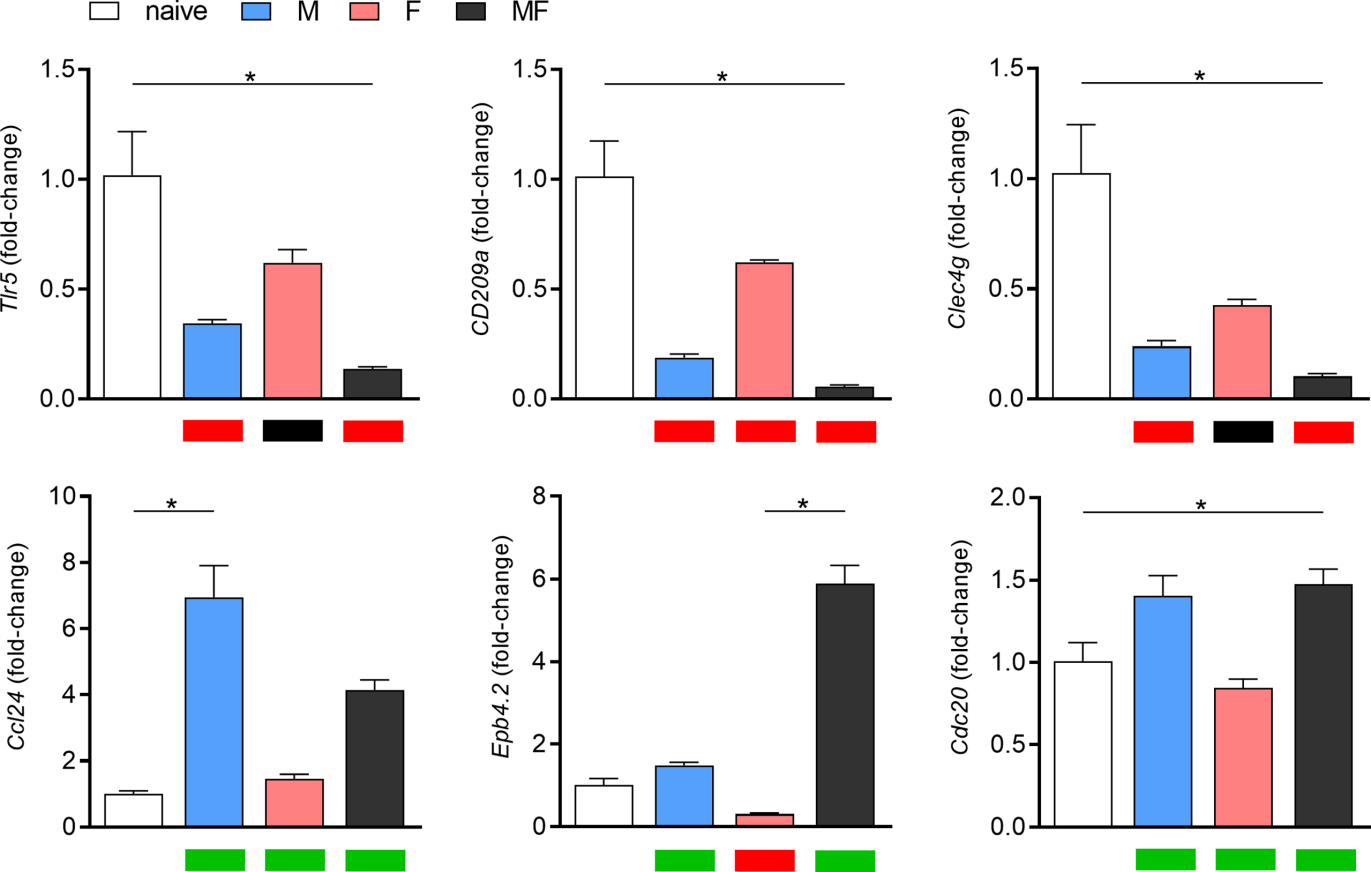
Validation of microarray data by RT-qPCR of six representative, highly regulated genes. Relative gene expression of *tlr5*, *cd209a*, *clec4g*, *ccl24*, *epb4.2*, and *cdc20* in spleens of *Schistosoma mansoni*-infected mice (M, F, MF) was determined by real-time PCR (8 wk p.i., *n* = 2–3). Real-time PCR data are expressed as fold-change and presented as bar graphs, while the corresponding microarray data are visualized as heat maps below the graphs (upregulation/green, downregulation/red, or unchanged/black). Real-time PCR data are presented as mean ± SEM. p values < 0.05 were considered statistically signi!cant. *p < 0.05; M, infected with male cercariae; F, infected with female cercariae; MF, infected with male and female cercariae.

To validate the microarray data, we determined the expression of six differentially regulated genes by real-time PCR (**Figure 4**). In general, gene expression levels were consistent with the results of the microarray analysis. Only with regard to cell division cycle 20 (*cdc20*) do our transcriptome data and the qPCR seem to diverge. The slight upregulation of *cdc20* shown in the heat map was not confirmed in the corresponding qPCR analysis.

In summary, the genes studied showed that processes involved in host immune response were strongly affected after bisexual and unisexual male infection, whereas unisexual female infection had little effect on the host immune system. Moreover, the immunoregulatory processes in the host after infection with *S. mansoni* appear to be triggered not only by the eggs but also by the adult worms *per se*.

### Infection With Male *Schistosoma mansoni* Leads to a Decrease in Splenic CD4^+^ T Cells and an Increase in Mature Dendritic Cells

**Figure 5:**
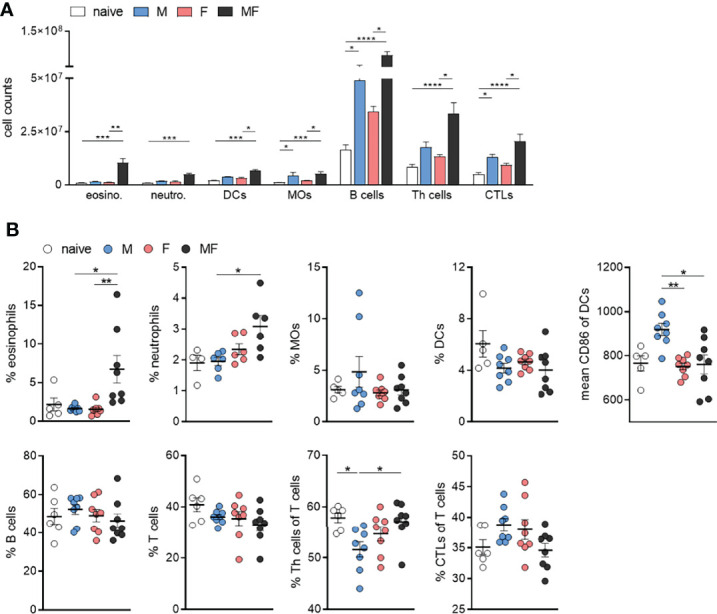
Infection with male *Schistosoma mansoni* leads to an increase in splenic CD86^+^ dendritic cells and a decrease in splenic CD4^+^ T cells. **(A)** Total numbers and **(B)** percentages of eosinophils, neutrophils, macrophages (MOs), dendritic cells (DCs), B cells, T cells, Th cells, and cytotoxic T cells as well as the proportion of CD86 on dendritic cells were analyzed by flow cytometry in homogenized spleen tissue of infected (M, F, and MF) and control (naive) mice (8 wk p.i., *n* = 4–8). Data are presented as mean ± SEM. p values < 0.05 were considered statistically significant. *p < 0.05, **p < 0.01, ***p < 0.001, ****p < 0.0001; M, infected with male cercariae; F, infected with female cercariae; MF, infected with male and female cercariae.

To analyze the composition of inflammatory immune cells in the spleen after unisexual and bisexual infection with *S. mansoni*, we performed flow cytometry on fresh spleen cell suspensions. The total cell number of immune cells analyzed was significantly increased in bisexually infected mice (MF) compared with naive mice. Compared with the MF group, we found significantly lower numbers of lymphoid and myeloid immune cells in the unisexually infected M and F groups (**Figure 5A**). The percentage of eosinophilic and neutrophilic granulocytes was significantly increased in group MF mice compared to unisexually infected mice (**Figure 5B**). The percentage of macrophages and dendritic cells in the spleen did not differ between the groups. The expression of CD86 on dendritic cells was significantly increased in group M compared to group F and MF. The percentage of B and T cells in the spleen did not seem to be affected by infection in our setting. However, in group M, the percentage of CD4^+^ T cells was significantly lower compared to naïve and MF mice, while group MF mice had a lower percentage of CD8^+^ T cells in the spleen compared to group M and F mice (**Figure 5B**). Taken together, the results suggest that the recruitment of granulocytes to the spleen is triggered by the eggs rather than the worm itself. Furthermore, our data suggest that male worms drive the maturation of dendritic cells (DCs) and induce T cell differentiation by upregulating the costimulatory molecule CD86.

### Splenocytes From Unisexually Infected Mice Produce Both Th1 and Th2 Cytokines Upon SWAP Stimulation

**Figure 6:**
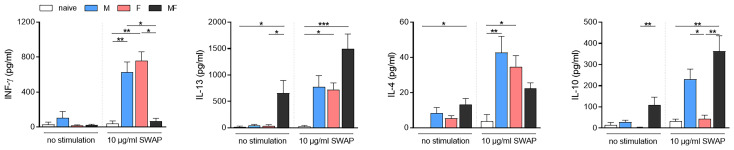
Unisexual infection with *Schistosoma mansoni* leads to a non-polarized Th1/Th2 immune response. Splenocytes were isolated from spleens of unisexually (M, F) and bisexually infected (MF) mice as well as healthy controls. Isolated splenocytes were stimulated with 10 μg/ml soluble worm antigen preparation containing eggs (SWAP). Supernatants were collected after 72 h and amounts of INF-γ, IL-13, IL-4, and IL-10 were quantified using ELISA (*n* = 5–8). Data are presented as mean ± SEM. *p* values <0.05 were considered statistically significant. *p < 0.05, ** p< 0.01, ***p < 0.001. M, infected with male cercariae; F, infected with female cercariae; MF, infected with male and female cercariae.

To specify the polarization of the splenic T cell response, we examined cytokine production by the splenocytes upon soluble worm/egg antigen (SWAP) stimulation (**Figure 6**). Secretion of INF-γ was significantly induced upon stimulation in unisexually infected mice (M and F), but not in bisexually infected individuals (MF), compared to the naive control. With regard to Th2-associatedcytokines, we determined significantly increased amounts of IL-13 in the supernatants of splenocytes from mice in group MF even without stimulation, compared to naive control mice. Splenocytes in groups M and F responded with IL-13 production to SWAP stimulation, but only half as much as in group MF. IL-4 production was highest in splenocytes of group M mice. Regulatory IL-10 was increased in the supernatants of SWAP-stimulated splenocytes in groups M and significantly for group MF, but not in group F. These data demonstrate that adult male and female *S. mansoni* are able to induce Th1 and Th2 immune responses in an egg-independent manner. Furthermore, IL-10 secretion appeared following SWAP stimulation of splenocytes in M and MF group mice, but not in group F.

## Discussion

Comparative transcriptomic and flow cytometric analysis of spleen tissue from unisexually infected mice (M and F) and naturally (bisexual, MF) infected mice revealed a total of 1,293 differentially regulated genes in group M, 512 genes in group F, and 4,062 genes in group MF compared to naive controls. The majority of the regulated genes were found in group MF, with a high level of correspondence to group M and only a small overlap with the regulated genes in group F. At the molecular level, Gene Ontology analyses revealed that processes involved in the host immune response were upregulated following infection with female worms, whereas host immune system processes were downregulated following bisexual infection or infection with male worms. At the cellular level, our data suggest that male worms drive the maturation of dendritic cells (DCs) and induce T-cell differentiation by upregulating the costimulatory molecule CD86. However, the percentage of CD4^+^ T cells was reduced in spleens of the group M mice. Furthermore, upon stimulation with SWAP, splenocytes from unisexually infected mice did not respond in a polarized manner, but secreted Th1 and Th2 cytokines.

Paired adult worms of *S. mansoni* live in the mesenteric veins of their host, where each pair lays up to 300 eggs daily (Moore and Sandground, 1956). The eggs are considered the most immunogenic parasite stage, eliciting a strong Th2 response in the host which is caused primarily by egg antigens (Grzych et al., 1991; Schramm and Haas, 2010). We identified a total of 4,062 genes that were differentially expressed in the spleens of MF-infected mice compared with naive controls (**Figure 2B**). Of these, 2,972 genes are regulated only in group MF. In contrast, we found a mere 157 regulated genes in group F and 231 in group M. It is likely that the high number of regulated genes in the MF group is due to the eggs produced by the worm pairs, as they have a high immunomodulatory potential (Schwartz and Fallon, 2018). Unisexual infection is therefore a good way of demonstrating the immunomodulatory effects of the adult worms without this being overridden by the highly immunogenic eggs. The limitation of this model is that it forces us to exclude any potential synergistic or counteracting effects of the worm pairs, as we can only examine them individually. Unfortunately, as far as we know, there is no model that allows the immunomodulatory effect of mated worms to be analyzed without eggs being laid.

Female schistosomes that develop in bisexual infections lie hidden in the gynecological duct of males, with whom they are in constant physical contact. In unisexual infections, on the other hand, females live free and unprotected from their hostile environment in the host’s bloodstream. Unisexual, unpaired worms, whether male or female, can survive for a very long time in the host. Recent publications demonstrate long survival of unpaired worms in the final host beyond 8 weeks up to 1 year (Lu et al., 2018; Lu et al., 2021). We identified 512 genes that are expressed differently in the spleens of group F mice compared to naive controls (**Figures 2B, C**). The low number of regulated genes in group F, compared to group M and MF, might be explained by the low degree of maturation of the female worms, on the one hand side (Fitzpatrick and Hoffmann, 2006). Moreover, the lower number of tegument proteins in female worms, identified *via* comparative proteomic analyses of male and female schistosomes, may indicate a lower interaction of female worms with the host (Winkelmann et al., 2022), which in turn results in reduced gene regulation in the host. Previous studies have generated contradictory results regarding the effects of female schistosomes on their host. On the one hand, unisexual infections with female cercariae increased the antibody response in baboons (Smithers, 1962). On the other hand, our own previous results have shown female schistosomes to have an immunosuppressive effect on the host (Koslowski et al., 2017; Sombetzki et al., 2018). In mice, infection with female schistosomes only was shown to produce fewer antigens than infection with male schistosomes only, although more antigens from female than male schistosomes were detected *in vitro* (Van Dam et al., 1996; Dumont et al., 2007).

The development of male schistosomes seems to be independent of the presence or absence of female worms, as unisexual and mated males show no morphological differences. When female worms are present, they are embraced by the males, which provide them with nutrients. The male worms transport the females to the oviposition site and are responsible for fertilization and maturation of the females (LoVerde et al., 2004). In this study, 1,293 genes were identified that were differentially expressed in the spleens of mice infected with male cercariae compared to naive controls (**Figure 2B**). This is more than twice the number of genes regulated by females (512 genes). The higher number of regulated genes in the spleen after unisexual infection with males suggests that males have a greater impact on their host in general. Our data are supported by previous studies demonstrating that male schistosomes induce a higher level of genetic diversity linked to antigenic polymorphism (Beltran et al., 2011), higher immunogenicity and higher pathogenicity compared to female schistosomes (Mota-Santos et al., 1977; Boissier et al., 1999). Thus, male schistosomes trigger strong innate immune reactions which lead to a reduction in worm and egg burden in the liver (Sombetzki et al., 2018). In addition, human participants exposed to male *S. mansoni* cercariae were described to develop Katayama syndrome (Langenberg et al., 2019).

Gene ontology analysis of upregulated genes following bisexual infection or infection with male schistosomes showed an enrichment for biological processes associated with the cell cycle, chromosome organization, and DNA packing (**Figure 3**). The genes discovered include *cdc20*, coding for cell division cycle protein 20 homologue, which is of extreme importance in cell cycle control and cell proliferation (Kapanidou et al., 2017). This might explain the higher spleen cell counts observed in groups MF and M (**Figure 1B**). Genes related to erythrocyte maturation and integrity are also upregulated in group M and MF mice compared with group F mice (**Figure 3** and **Supplementary Table 1**; Perkins et al., 1995; Satchwell et al., 2009; Cantù et al., 2011). Male schistosomes consume ~100 nl per day and female ~900 nl per day (Skelly et al., 2014). The higher feeding rate of female worms is related to egg production in the mated state. The blood consumption rate of females in unisexual infection is not yet known, but is certainly far below that of the other worms due to their smaller body size. Both group F and group M mice show an upregulation of antibody-related genes. However, in group M, in addition to a gene encoding the Fc receptor (fcamr), the receptors for IgG are also upregulated compared to the control mice. This could be indicative of higher antigen diversity on the part of the male worms. Other upregulated genes in group M mice, such as *apol10a*, *apol11a*, or *apol11b* (**Figure 3B**), encode apolipoproteins. Apolipoproteins are a protein component of lipoproteins and circulate in the blood transporting hydrophobic lipids such as tryacylglycerols (TGs) and cholesterol. It has been shown that, unlike in adult female schistosomes, TGs are the dominant lipid species on the surface of adult male schistosomes (Kadesch et al., 2020). There is evidence that lipoproteins are not only responsible for lipid transport between tissues and organs, but are also involved in immune defense against parasites (Han, 2010). In addition, adult schistosomes rely on the uptake of cholesterol and fatty acids from the host to form species-specific lipids with potentially immunomodulatory properties (Van Hellemond et al., 2006; Giera et al., 2018). The *ccl24* gene was also strongly upregulated in group M mice. Chemokine (C-C motif) ligand 24 is a small cytokine chemotactic for eosinophils, resting T lymphocytes and neutrophils (Patel et al., 1997; White et al., 1997). The plasma concentration of CCL24 was shown to be significantly higher in *S. mansoni*-infected individuals than in non-infected individuals (Rodrigues Oliveira et al., 2018). Higher expression of CCL24 is one more indicator of the stimulation of the host immune system by male worms. Moreover, INF-γ and interferon-stimulated genes were also found to be most upregulated in group M mice (**Figure 3** and **Supplementary Table 1**). These results are made even more striking by the fact that we also see significant IL-10 production after stimulation of splenocytes, which is known to suppress INF-γ (**Figure 6**; Flores Villanueva et al., 1994; Mosman, 1994).

Downregulated genes following infection with male schistosomes were found to be involved in cellular processes such as signaling and adhesion (**Figure 3A**). The proteins affected include the pattern recognition receptors (PRRs) of antigen-presenting cells (APC), including DC-SIGN (CD209) and toll-like receptor 5 (TLR5). Dendritic cell-specific ICAM-3-grabbing nonintegrin (DC-SIGN, CD209) can recognize schistosome glycoconjugates (Van Liempt et al., 2007), and as APCs, DCs are important for initiating adaptive immune responses. Some studies have indicated that schistosome components interact with TLRs, which are receptors on DCs (Van der Kleij et al., 2002; Thomas et al., 2003; Aksoy et al., 2005). Thus, male worms may be able to modulate DC function *via* downregulation of pattern recognition receptors on APCs. On the other hand, we found increased expression of CD86 on DCs in group M, while the percentage of DCs within spleens did not differ between groups and the percentage of CD4^+^ T cells was significantly lower in group M mice than in control mice and group MF (**Figure 5A**). CD86 is expressed on antigen-presenting cells (APCs) such as DCs and macrophages. It is a marker of early DC maturation and is also described to be a critical costimulatory molecule in the initial steps of T cell activation (Caux et al., 1994; Lim et al., 2012). Thus, although male worms seem to stimulate the expression of CD86 on DCs, at the same time we see a lower proportion of CD4^+^ T cells in the spleens of animals from the M group. This observation might be related to increased IL-4 and IL-10 production by the splenocytes of group M mice. In addition to Th1 immune response, we also found Th2-associated cytokines to be secreted by SWAP-stimulated splenocytes following single-sex infection with male or female schistosomes. Apparently, the lack of soluble egg antigens leads to an unpolarized immune response that manifests in splenocytes producing a mixture of Th1 and Th2 cytokines. As expected, there is little evidence of a Th1 response in the MF group, but high levels of Th2 cytokines such as the pro-fibrotic IL-13. However, higher levels of the Th2 cytokine IL-4 are found in the splenocytes of group M mice than in groups MF or F. The high IL-10 level also measured in this group underlines the importance of IL-4 in controlling IL-10 production and thus the capacity of male schistosomes to regulate the immune response independent of parasite eggs (Mitchell et al., 2017).

As mentioned above, infection with female worms has less influence on gene regulation than infection with male worms or worm pairs. Among the most upregulated genes we detected in group F were *activation-induced cytidine deaminase* (*aicda*) and *fcamr* (**Figure 3** and **Supplementary Table 1**). AICDA causes mutations in DNA that produce antibody diversity by initiating somatic hypermutation and class-switch recombination of immunoglobulin genes in B cells (Muramatsu et al., 2000). The gene for Fc receptors on antigen-presenting cells (APCs; macrophages, dendritic cells and B cells) binds IgA and IgM and is upregulated to bind these antibodies and initiate adaptive immune response. However, schistosomes have developed specific ways to circumvent this antibody-mediated immune response. They are able to bind immunoglobulins *via* their Fc receptor to mask themselves with host proteins (Torpier et al., 1979). They are also able to cleave immunoglobulins directly using serine proteases (Auriault et al., 1981; Pleass et al., 2000). Genes of interleukin-1β (IL-1β) and “Resistins” are also upregulated in group F (**Figure 3** and **Supplementary Table 1**). IL-1β, primarily produced by blood monocytes and tissue macrophages, mediates both acute and chronic inflammation (Bird et al., 2002). Resistin has been shown to have potent proinflammatory properties, playing an important role in triggering the release of other proinflammatory cytokines such as TNF-α, IL-1β, and IL-6 (Bokarewa et al., 2005). Furthermore, resistin-like molecule alpha (Retnla) functions as a negative regulator of Th2 responses (Pesce et al., 2009). Downregulated genes in group F were shown to be involved in erythrocyte homeostasis. Here, *epb4.2* stands out, which is downregulated in group F only (**Figure 3**; **Supplementary Table 1**). *Epb4.2* encodes a protein that is one of the most abundant and structurally important components of the peripheral erythrocyte membrane (Satchwell et al., 2009).

The lists of regulated genes presented here may be useful to decipher host–parasite interactions to identify targets for elimination for adult schistosomes. For example, ligands for lipoprotein uptake by adult schistosomes are a potential therapeutic target. Silencing the gene could first clarify the importance of this ligand for adult worm survival in the host. Also, immunization with the ligands as antigen could provide protection against infection. In addition, identification of potential parasite binding partners of TLRs as receptors on APCs or AICDA would also be of particular interest to identify potential targets.

Overall, our results suggest that male adult worms have a qualitatively diverse and strong immunogenic effect which induces an imbalanced Th1/Th2 immune response in the host, possibly affecting immune regulation. However, female worms also affect the gene expression profile in the spleen of mice, albeit to a lesser extent. Data collections of this kind are of great importance in understanding the effects of pathogens on the host organism and finding targets to repel or eliminate the invaders.

## Data Availability Statement

The data presented in the study is deposited in Gene Expression Omnibus (GEO) repository, accession number GSE197804.

## Ethics Statement

All animal experiments were performed in strict accordance with the German regulations of the Society for Laboratory Animal Science and the European Health Law of the Federation of Laboratory Animal Science Associations. The animal study was reviewed and approved by State Office for Agriculture, Foodsafety and Fisheries Mecklenburg-Western Pomerania (7221.3-2-022/17-3). All efforts were made to minimize the suffering of animals.

## Authors Contributions

FW, AR and MS designed the study. FW, AR, CS, and MS conducted experiments and analysed the data. FW, AR, NK, ECR, and MS contributed to data interpretation. DK conducted instrumental transcriptomic analysis, offered technical expertise and contributed to interpretation of data. FW and MS wrote the manuscript. All authors reviewed the manuscript.

## Funding

The work was founded by the European Social Fund project Card-ii-omics (grant number ESF/14-BM-A55-0037/16) and FORUN program of Rostock University Medical Center (grant number 889009).

## Conflict of Interest

The authors declare that the research was conducted in the absence of any commercial or financial relationships that could be construed as a potential conflict of interest.

## Publisher’s Note

All claims expressed in this article are solely those of the authors and do not necessarily represent those of their affiliated organizations, or those of the publisher, the editors and the reviewers. Any product that may be evaluated in this article, or claim that may be made by its manufacturer, is not guaranteed or endorsed by the publisher.
