# Supplementary material for: Sex-Specific Modulation of the Host Transcriptome in the Spleen of Schistosoma mansoni-Infected Mice
Source: Front Cell Infect Microbiol. 2022 Jul 5;12:893632. doi: 10.3389/fcimb.2022.893632 (PMC9294737; doi:10.3389/fcimb.2022.893632)
Supplement: Supplementary file 1 [file DataSheet_1.pdf]

Supplementary Table 1. List of regulated genes associated with host immune responses in the spleen of female C57BL/6J mice infected with male (M) or female (F) *Schistosoma mansoni* cercariae, or bisexually infected (MF) compared to naive control mice (8 wk p.i., n=2-3).

				MALE			FEMALE			MALE/FEMALE		
**Function related to:**	**Probeset ID**	**Gene Symbol**	**Description**	**Fold-change**	***p* value**	**FDR**	**Fold-change**	***p* value**	**FDR**	**Fold-change**	***p* value**	**FDR**
Antibodies	TC0600001386.mm.2	*Aicda*	activation-induced cytidine deaminase	-27.04	9.38E-07	6.00E-04	-20.42	2.38E-06	1.12E-02	-8.18	2.00E-04	1.90E-03
	TC0100001164.mm.2	*Fcamr*	Fc receptor, IgA, IgM, high affinity	-10.98	3.27E-06	1.10E-03	-11.35	2.82E-06	1.12E-02	-3.43	2.20E-03	1.40E-02
	TC0300002506.mm.2	*Fcgr1*	Fc receptor, IgG, high affinity I	-4.23	5.42E-07	5.00E-04	-2.13	1.00E-04	4.76E-02	-2.3	2.00E-04	1.90E-03
	TC0100001574.mm.2	*Fcgr4*	Fc receptor, IgG, low affinity IV	-10.38	1.03E-07	2.00E-04	-2.69	4.00E-04	8.66E-02	-4.81	1.51E-05	3.00E-04
	TC0100003584.mm.2	*Fcer1a*	Fc receptor, IgE, high affinity I, alpha polypeptide	-	-	-	-	-	-	-7.56	7.36E-06	2.00E-04
Apolipoproteins	TC1500001818.mm.2	*Apol8*	apolipoprotein L 8	-5.38	2.89E-05	3.50E-03	-	-	-	-60.53	1.22E-08	2.73E-06
	TC1500000627.mm.2	*Apol10a*	apolipoprotein L 10a	-30.32	2.80E-10	3.10E-06	-2.06	1.20E-03	1.25E-01	-222.11	3.98E-12	4.42E-08
	TC1500000628.mm.2	*Apol11a*	apolipoprotein L 11a	-87.66	1.31E-09	9.72E-06	-2.93	9.00E-04	1.13E-01	-460.78	1.07E-10	4.36E-07
	TC1500001817.mm.2	*Apol11b*	apolipoprotein L 11b	-41.34	1.73E-10	3.10E-06	-2.79	1.00E-04	4.55E-02	-368.78	3.72E-12	4.42E-08
Cell cycle	TC0400003390.mm.2	*Cdc20*	cell division cycle 20	-7.56	3.31E-06	1.10E-03	-2.52	2.70E-03	1.63E-01	-20.16	1.46E-07	1.11E-05
	TC0700004451.mm.2	*Mki67*	antigen identified by monoclonal antibody Ki 67	-4.62	1.63E-06	7.00E-04	-	-	-	-14.82	9.20E-09	2.35E-06
Mediators	TC0500003399.mm.2	*CCL24*	chemokine (C-C motif) ligand 24	-25.34	4.40E-07	5.00E-04	-2.06	3.47E-02	3.73E-01	-19.04	7.17E-06	2.00E-04
	TC0200004602.mm.2	*IL1b*	interleukin 1 beta	-	-	-	-4.49	1.00E-05	1.31E-02	-	-	-
	TC0100000298.mm.2	*Il1rl1*	interleukin 1 receptor-like 1	-	-	-	-	-	-	-21.85	8.54E-08	7.75E-06
Erythrocytes	TC0200004475.mm.2	*Epb42*	erythrocyte membrane protein band 4.2	-3.61	6.51E-05	5.80E-03	4.51	3.43E-05	2.18E-02	-33.92	8.01E-09	2.21E-06
	TC0800000971.mm.2	*Klf1*	Kruppel-like factor 1 (erythroid)	-3.14	3.00E-04	1.29E-02	2.26	7.00E-03	2.25E-01	-34.39	5.07E-08	5.84E-06
	TC1700000817.mm.2	*Rhag*	Rhesus blood group-associated A glycoprotein	-3.15	7.28E-05	6.10E-03	4.31	2.87E-05	1.93E-02	-41.07	4.59E-09	1.91E-06
	TC0700004148.mm.2	*Sox6*	SRY (sex determining region Y)-box 6	-6.62	5.22E-06	1.30E-03	-	-	-	-60.14	1.84E-09	1.24E-06
Granulocytes	TC0300001704.mm.2	*Cpa3*	carboxypeptidase A3, mast cell	-2.11	2.09E-02	1.67E-01	-	-	-	-23.77	8.00E-07	3.44E-05
	TC1400001868.mm.2	*Ear1*	eosinophil-associated, ribonuclease A family, member 1	-2.21	1.85E-02	1.55E-01	-	-	-	-182.61	1.01E-09	9.19E-07
	TC1400000594.mm.2	*Ear7; Ear6*	eosinophil-associated, ribonuclease A family, member 7 & member 6	-2.41	3.49E-02	2.19E-01	-	-	-	-338.05	8.08E-09	2.21E-06
	TC1000000823.mm.2	*Elane*	elastase, neutrophil expressed	-	-	-	-	-	-	-11.31	1.14E-06	4.38E-05
	TC1100003488.mm.2	*Epx*	eosinophil peroxidase	-	-	-	-	-	-	-266.11	3.08E-09	1.51E-06
	TC1400002113.mm.2	*Mcpt8*	mast cell protease 8	-2.12	6.00E-04	1.98E-02	-	-	-	-80.07	2.27E-10	6.29E-07
	TC0900001461.mm.2	*Ngp*	neutrophilic granule protein	-	-	-	2.97	1.12E-02	2.66E-01	-11.6	4.68E-06	1.00E-04
	TC0200001227.mm.2	*Prg2*	proteoglycan 2, bone marrow	-	-	-	-	-	-	-134.58	3.36E-10	6.79E-07
Interferons	TC0300001447.mm.2	*Gbp2*	guanylate binding protein 2	-8.82	2.79E-07	4.00E-04	-3.63	1.00E-04	4.78E-02	-	-	-
	TC0300001444.mm.2	*Gbp2b; Gbp5*	guanylate binding protein 2b; guanylate binding protein 5	-3.88	6.62E-06	1.50E-03	-	-	-	-	-	-
	TC0500002895.mm.2	*Gbp10; Gbp6*	guanylate-binding protein 10; guanylate binding protein 6	-6.37	2.14E-06	9.00E-04	-2.76	5.00E-04	9.11E-02	-	-	-
	TC1100002643.mm.2	*Gm12185; Tgtp1*	predicted gene 12185; T cell specific GTPase 1	-9.55	3.73E-08	1.00E-04	-3.83	1.29E-05	1.44E-02	-4.62	4.67E-06	1.00E-04
	TC1000001452.mm.2	*Ifng*	interferon gamma	-9.92	4.66E-07	5.00E-04	-2.59	7.30E-03	2.28E-01	-2.35	5.40E-03	2.89E-02
	TC1800000610.mm.2	*Iigp1*	interferon inducible GTPase 1	-11.84	6.09E-08	2.00E-04	-4.26	1.89E-05	1.56E-02	-3.65	1.00E-04	1.40E-03
	TC1100002649.mm.2	*Tgtp2*	T cell specific GTPase 2	-8.27	7.16E-08	2.00E-04	-3.7	9.90E-06	1.31E-02	-4.98	3.25E-06	9.28E-05
Pattern recognition receptors	TC0800001619.mm.2	*Cd209a*	CD209a antigen	3.84	2.00E-04	9.80E-03	-	-	-	5.61	4.39E-05	6.00E-04
	TC0800001624.mm.2	*Cd209b*	CD209b antigen	5.28	3.84E-06	1.20E-03	2.66	4.00E-04	9.11E-02	5.11	1.01E-05	2.00E-04
	TC0800001617.mm.2	*Clec4g*	C-type lectin domain family 4, member g	3.67	3.68E-05	4.00E-03	-	-	-	15.17	7.54E-08	7.34E-06
	TC0100001754.mm.2	*Tlr5*	toll-like receptor 5	4.60	5.16E-06	1.30E-03	-	-	-	3.99	1.25E-05	3.00E-04
Resistins	TC0800000019.mm.2	*Retn*	resistin	-	-	-	-2.39	2.97E-02	3.55E-01	-	-	-
	TC1600000698.mm.2	*Retnla*	resistin like alpha	-	-	-	-3.84	3.05E-02	3.58E-01	-2.49	4.20E-02	1.42E-01
	TC1600000699.mm.2	*Retnlg*	resistin like gamma	-6.19	1.00E-04	8.30E-03	-9.31	1.00E-04	4.35E-02	-11.18	2.45E-05	4.00E-04
